# Supplementary material for: Neural representations of situations and mental states are composed of sums of representations of the actions they afford
Source: Nat Commun. 2024 Jan 19;15:620. doi: 10.1038/s41467-024-44870-7 (PMC10799018; doi:10.1038/s41467-024-44870-7)
Supplement: Supplementary file 1 — Supplementary Information [file 41467_2024_44870_MOESM1_ESM.pdf]

SUPPLEMENTARY MATERIALS

Summation selectivity analysis

For the confirmatory summed state hypotheses that were corroborated, we registered a follow-up exploratory analysis to investigate the role of affordance selectivity. Specifically, we hypothesized that actions with more selective affordance profiles – that is, actions that occur in a relatively specific set of situations or mental state – would play a large role in defining those situations/states. To test this, we computed a selectivity index from the online rating data. For each action, we compute the difference between the average and the maximum co-occurrence likelihood rating across all situations, or across all actions. In parallel, we used the fMRI data to compute a reconstruction contribution index. To do this, we compute repeated the pattern summation analyses 60 times, leaving out each action in turn. We compared the reconstruction accuracy in each of these cases to the reconstruction accuracy using all 60 actions. The size of the difference between these values was taken as an indicator of how much each action contributed to defining situations/mental states. Finally, we correlated the selectivity index with the reconstruction contribution index to determine if actions with more selective affordances contributed more to reconstructing neural representations of social situations or mental states. We did not detect a statistically significant correlation in either situation or state reconstruction.

Pattern summation model selection

The primary pattern summation analyses reported in the main text provide direct evidence that affordance-weighted sums of action representations can reconstruct situation and mental state representations. However, what proportion of actions contribute to the representations of any given situation or state? In principle, the pattern summation results are consistent with the possibility that only one action (e.g., the most likely) pattern is correlated

with a given situation pattern, with the rest of the actions contributing only noise to the reconstruction of that situation pattern. If so, this would contradict our hypothesis that situation and mental state representations reflect sums of the actions they afford.

To address this possibility, we conducted an exploratory variation of the pattern summation analyses. Using cross-validated model selection, we estimated the proportion of actions that contribute to each situation and each state. This analysis proceeded through the following steps. First, for a given situation, we concatenated all participants' corresponding patterns of brain activity in a single vector. These patterns were first z-scored to account for differences in baseline activity between participants. Second, we likewise concatenated all participants' action patterns for each of the 60 actions. Third, we regressed the situation patterns onto the action patterns. This regression was fit using LASSO, in ordered to facilitate feature selection. This process was repeated across four cross-validation folds, training the LASSO regression on 21/28 participants on each fold, and testing on the held out 7. Performance in the test set was used to determine the optimal lambda parameter of the LASSO. Based on that optimal lambda, we could compute how many non-zero coefficients (not counting the intercept) remained in the regression. This provided us with an estimate of how many actions contribute to optimally reconstruct each situation. We repeated this process using each of the 60 situations as the dependent variable, and then each of the 60 mental states. We also repeated the entire process 10 times with different random cross-validation folds to ensure the stability of the results.

After completing this procedure, we calculated the median number of non-zero regression coefficients for each situation/state across the 4 cross-validation folds and the 10 replicates. This yielded estimates of how many of the 60 action patterns contributed to each of the 60 situations/states. These estimates ranged from 17.5 to 39 actions for situation reconstruction,

with a mean of 26.46 actions contributing to each situation; for mental states, these estimates ranged from 16 to 38 actions, with a mean of 24.12 actions contributing to each state. To perform inference from the sample of situations/states we examined to the broader populations thereof, we applied percentile bootstrapping to these estimates, converted into percentages of out 60 actions. The 95% confidence interval for situations ranged from 42.3% to 45.9% of actions contributing to the average situation, and the interval for mental states ranged from 38.5% to 42.0% of actions contributing to the average state.

The results of this cross-validated model selection procedure suggest that many actions contribute to represent the representation of a given situation or mental state. However, are these contributions in proportion to how much those situations/states afford those situations, as we hypothesized? To answer this question, we conducted a variant of the primary representational similarity analyses reported in the main text. Specifically, we fit LASSO regressions predicting each participants' situation/state pattern using their action patterns, with the lambda parameter set using the mean of the optimal lambda's estimated during the model selection process described above. This yielded 60 beta estimates – one for each action – for each of the 60 situations/states for each participant. We combined these into a single 60 (situation) x 60 (action) neural weight matrix for each participant, analogous to the neural similarity matrices used in the main representational similarity analyses. For each participant, we vectorized their neural weight matrix, and correlated it with the corresponding average action affordance matrix provided by the online participants. We z-scored the action affordance matrix with respect to actions first, to account for the fact that the neural weight matrix reflected the results of separate regressions. The resulting correlations were Fisher-transformed and entered into one-sample t-tests against the null hypothesis of zero average correlation. We found statistically significant correlations

between the situation-action neural weight matrix and affordance matrix (mean $Z(r)$ = .019, d = 1.06, $p_{corrected}$ = 5.6 x 10^{-6}), and between the mental state-action neural weight matrix and affordance matrix (mean $Z(r)$ = .016, d = .91, $p_{corrected}$ = 4.8 x 10^{-5}). These results thus replicate the results of the primary representational similarity analysis. Altogether, the cross-validated model selection pattern summation analysis and subsequent representational similarity analysis show that: i) many actions contribute to the representation of each situation and mental state, and ii) those contributions are proportional to the situations/states action affordances. These results thus further corroborate the overall hypotheses of the investigation.

Representational similarity analysis with Kendall's τ

As a robustness check on the representational similarity analyses reported in the main text, we re-ran these analyses using Kendall's τ rank correlation coefficients instead of Pearson correlations to compare the neural pattern similarity matrices and the co-occurrence rating matrices. Since Kendall's τ does not have the same distribution as the Pearson's r, we did not apply Fisher's r-to-z transform prior to computing statistical significance. We again found that co-occurrence ratings predicted neural pattern similarity in all three cases, including between actions and situations (mean τ = .035, d = .90, $p_{corrected}$ = .00030), between actions and mental states (mean τ = .038, d = 1.80, $p_{corrected}$ = .000010), and between situations and mental states (mean τ = .016, d = .61, $p_{corrected}$ = .010). This outcome indicates that the representational similarity analysis results are robust to choice of correlation coefficient.

Within-domain representational similarity analyses

The primary pattern summation analyses and representational similarity analyses indicate that situations and mental state representations are composed of sums of the representations of actions they afford. These analyses directly compared patterns of brain activity corresponding to

different situations/states to patterns of brain activity corresponding to different actions. By doing so, these analyses provide strong evidence that situation/state representations are literal voxelwise sums of action representations. However, this analysis is limiting, in that it can only address representations of situations and mental states in regions where they overlap with action representations.

To examine whether action affordances shape situation and mental state representations across the full set of voxels that encode situations/states, we conducted a pair of additional exploratory representational similarity analyses. To this end, we generated conventional (i.e., within-domain, symmetric) neural pattern similarity matrices by correlating all situations patterns with all other situation patterns, and all mental state patterns with all other mental state patterns. The patterns used to generate these matrices were taken from the full set of voxels representing situations, and the full set of voxels representing mental states, respectively, rather than just the voxels overlapping with action representations. We computed corresponding model similarity matrices by taking the (reverse-coded) Euclidean distances between the situations' action affordances, and the mental states' action affordances, respectively. We vectorized participants neural similarity matrices and correlated them with the likewise vectorized model matrices. The resulting Pearson correlation coefficients were Fisher transformed and entered into one-sample t-tests against the null of zero average correlation. We observed a significant correlation between neural pattern similarity and action affordance similarity with respect to situations (mean $Z(r) = .054$, $d = 1.035$, $p_{\text{corrected}} = 8.4 \times 10^{-6}$) but not with respect to mental states (mean $Z(r) = -.017$, $d = .36$, $p_{\text{corrected}} = .069$). These results suggest that, even beyond regions of overlapping situation and action representation, action affordances predict how

situations are represented. However, the relationship between mental state representation and action affordances may be limited to regions of overlapping state and action representation.

Estimating the shape of the action-weighting function

In our preregistered analyses, our hypotheses assumed that the summation of action representations to form situation or state representations consisted of a linear weighted average. That is, the least afforded actions would take on weights close to zero, and more afforded actions would take on weights linearly proportional to their co-occurrence with the situation/state in question. However, this is not the only weighting pattern that could make sense. For example, the brain could place a disproportional amount of weight on the most likely actions or assign negative weights to unlikely actions. To address these possibilities, we conducted exploratory analyses based on the representational similarity analyses reported in the main text.

In these analyses, we decomposed the co-occurrence rating matrices into 60 parts. Each of these 60 60 x 60 matrices represented the Nth most likely action for each situation/state. So, for example, the first of these matrices consisted of matrix of all zeros, except for a single “1” in each row, corresponding to the most likely action to occur in that situation/state. We fit multiple regressions with neural pattern similarity matrices (situation-action or state-action) as the dependent variables, and the 60 binary matrices (1st through 60th most likely actions) as the independent variables. We tested the statistical significance of all 60 independent variables across participants using the same approach we applied in the main RSAs.

The results of this analysis (Figure S7) addressed the two important issues. First, we observed that no one action – not even the most likely one – was sufficient to account for the overall effect of co-occurrences on neural pattern similarity. In both the situation-action and

state-action cases, multiple actions were significant predictors of the neural RDM. This corroborates our hypothesis that situations and mental states are sums of action affordances.

Second, the results offer an even more precise way of describing the relation between actions and situations/states. By fitting a loess curve to the effect sizes of the 60 action models, we obtained a smooth estimate of the shape of the optimal co-occurrence weighting function. The shape of this function was similar across both the situation-action and state-action cases. In both cases, it was clearly nonlinear. Specifically, we observed that highly likely actions were disproportionately highly weighted, relative to what a linear weighting would predict. Additionally, some of the weights were significantly negative. This suggests that situations/states are defined not only by the sum of actions they do afford, but also in the negative by the sum of actions they do not afford.

SUPPLEMENTARY FIGURES

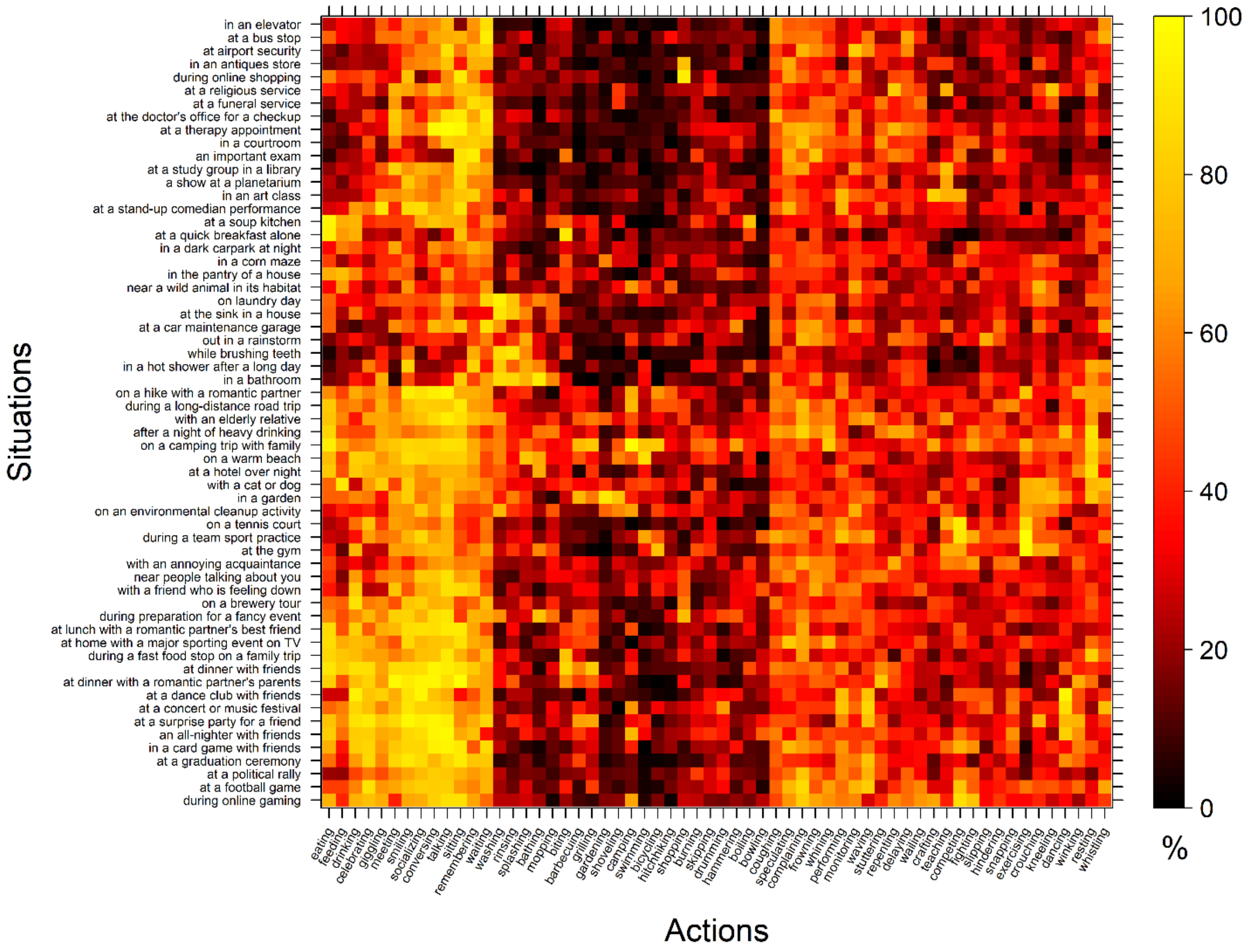

Figure S1. The heatmap visualizes ratings of the likelihood of each action (x-axis) occurring in each situation (y-axis) among the final sets of 60 situations and 60 actions used as stimuli in the fMRI experiment. The ratings shown were provided by an online sample and averaged across participants (10 per cell on average). Warmer colors indicate higher average probability ratings.

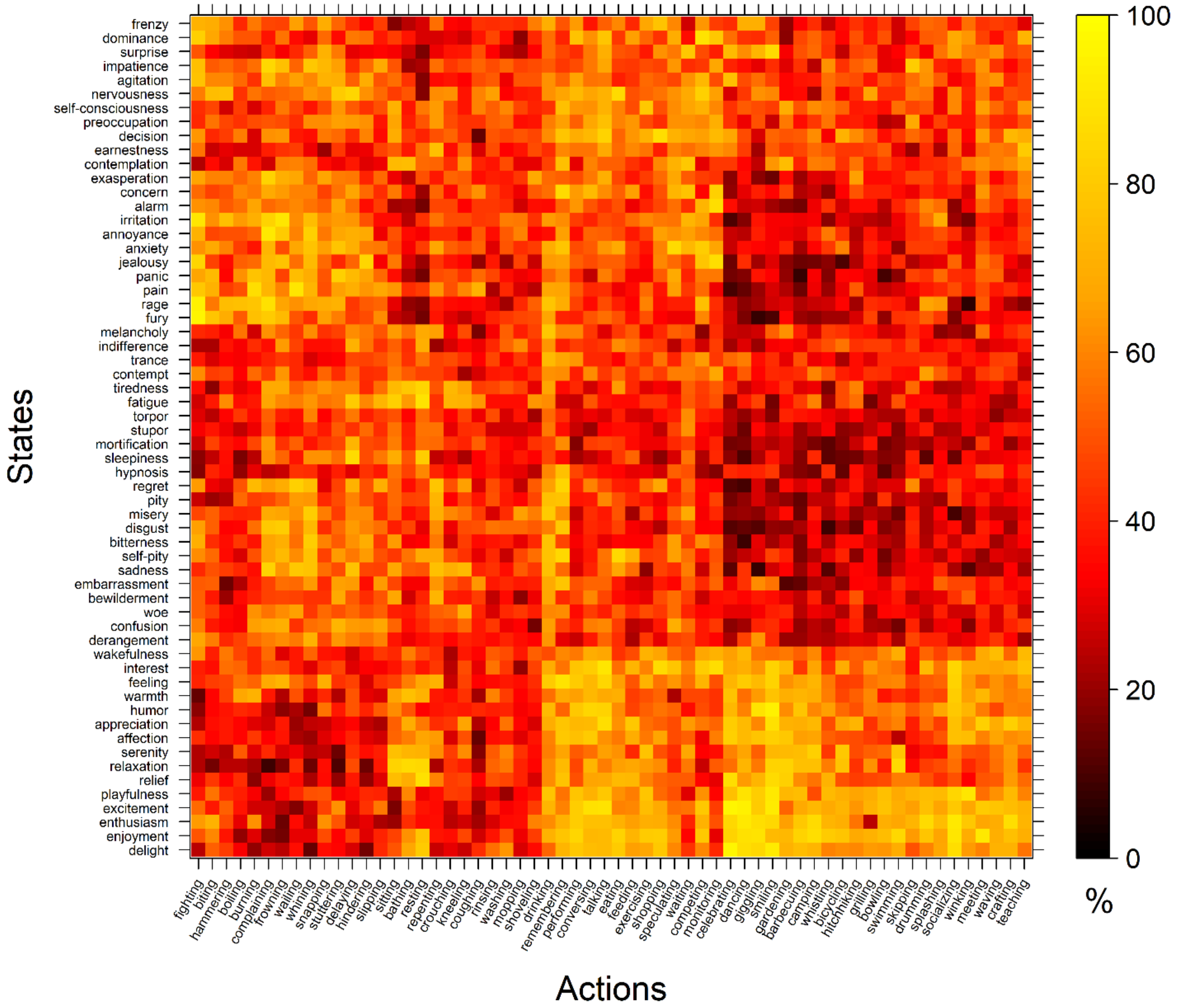

Figure S2. The heatmap visualizes ratings of the likelihood of each action (x-axis) occurring in each mental states (y-axis) among the final sets of 60 situations and 60 states used as stimuli in the fMRI experiment. The ratings shown were provided by an online sample and averaged across participants (10 per cell on average). Warmer colors indicate higher average probability ratings.

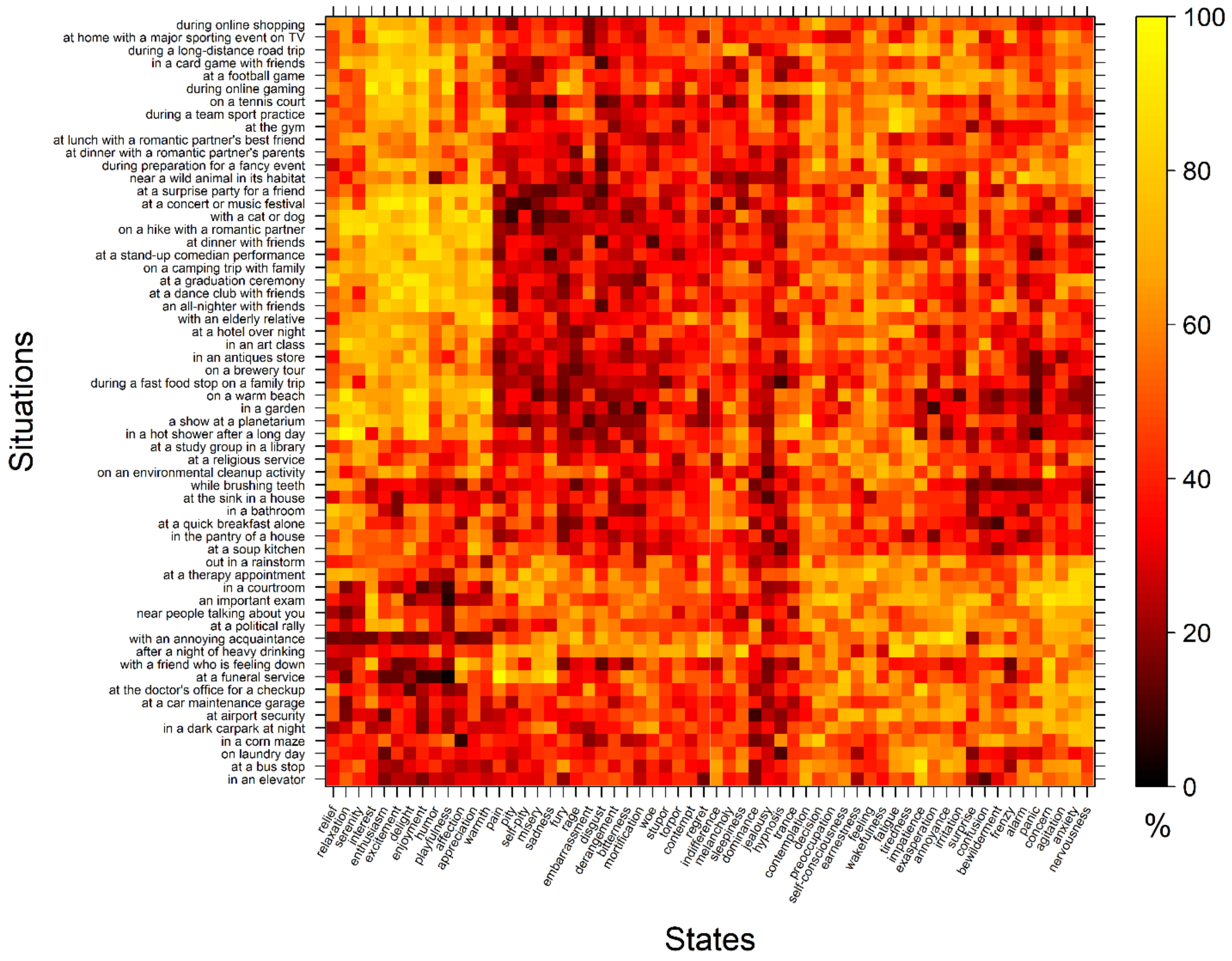

Figure S3. The heatmap visualizes ratings of the likelihood of each mental state (x-axis) occurring in each situation (y-axis) among the final sets of 60 mental states and 60 situations used as stimuli in the fMRI experiment. The ratings shown were provided by an online sample and averaged across participants (10 per cell on average). Warmer colors indicate higher average probability ratings.

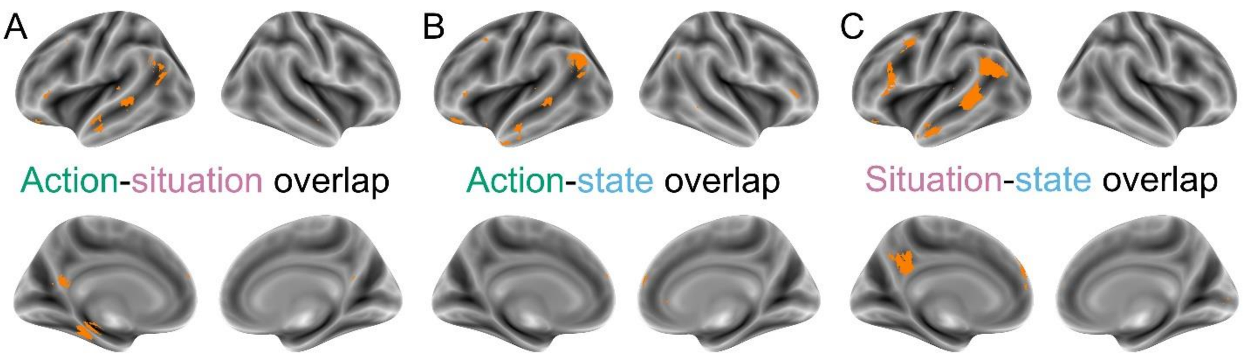

Figure S4. Orange regions reflect areas of overlap between voxels selected for their reliability in representing A) actions and situations, B) actions and states, and C) situations and states. Word colors represent the class of stimulus, as in other figures: actions – green, mental states – blue, and situations – pink.

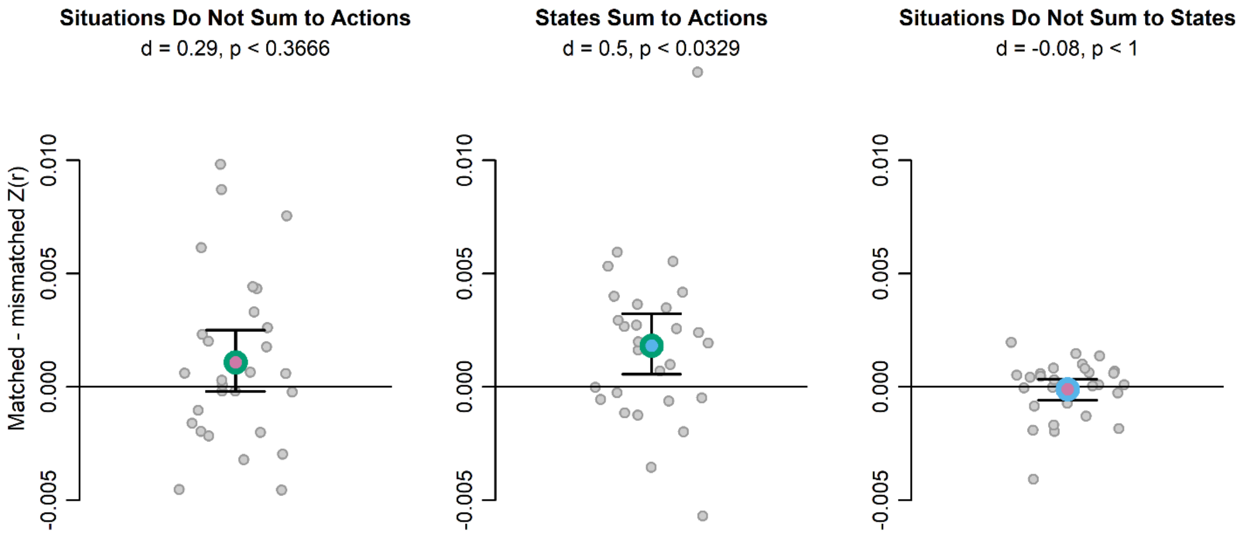

Figure S5. Preregistered exploratory pattern summation analyses tested the reverse directions of these hypotheses, finding that mental state patterns sum to action patterns, but situation patterns sum to neither action patterns nor mental state patterns. Colors represent the classes of stimuli involved in each test, as in other figures: actions – green, mental states – blue, and situations – pink. Reported p-values were derived from permutation testing on two-tailed, one-sample t-tests, with familywise error rate controlled via the maximal statistic (n = 28 participants). Y-axis values reflect differences in Fisher z-transformed [Z(r)] pattern correlations between matched and mis-matched patterns. Error bars represent 95% bootstrapped confidence intervals around the mean. Individual subjects are shown in grey.

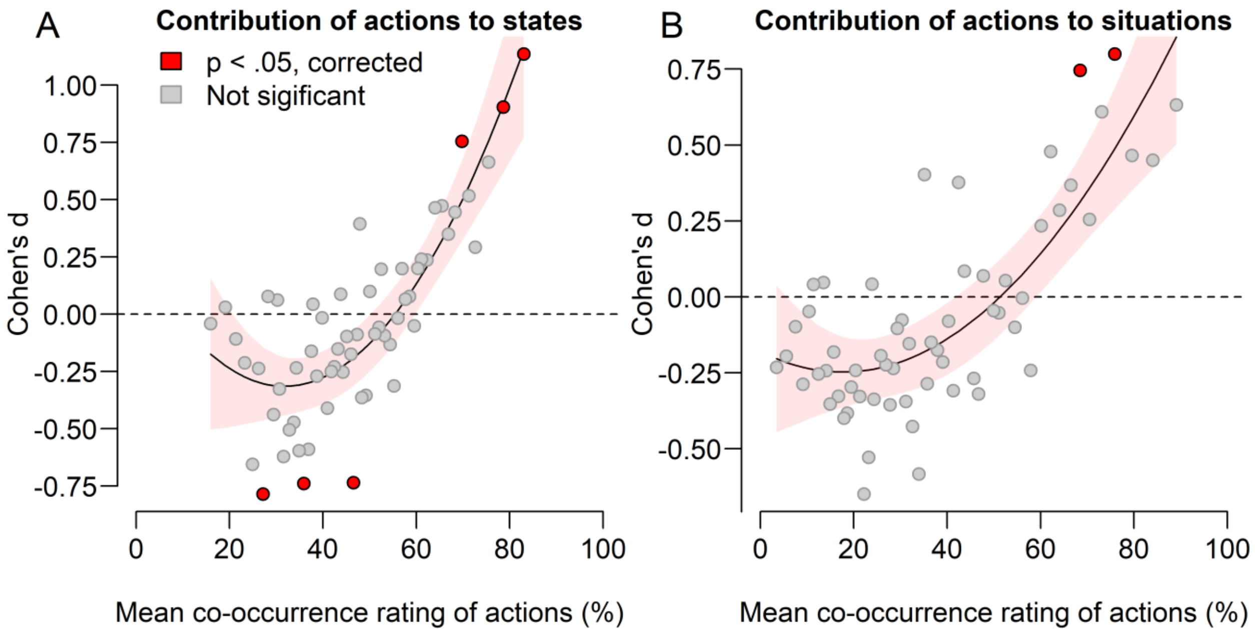

Figure S6. Representational similarity analyses estimate how much the 1st through 60th most likely action in each (A) mental state and (B) situation contributes to explaining pattern similarity. The results indicate that mental states and situations are nonlinear functions of the actions they afford, and are defined not only by the actions they do afford, but also in the negative by those they do not. The x-axes indicate the average co-occurrence ratings for the least likely action (left-most point) through the most likely action (right-most point). The y-axes indicate the mean neural pattern similarity between states/situations and each action rank. Red points indicate action ranks that significantly predict neural similarity in two-sided one-sample t-tests ($p < .05$, controlling for multiple comparisons via maximal statistic permutation testing) whereas grey points were not statistically significant. Loess curves with 95% Bonferroni-corrected confidence intervals (red) provide a smooth estimate of the optimal weighting of action patterns to reconstruct state/situation patterns. Participant n = 28; stimulus n = 60 of each type.
