# Supplementary material for: Neural representations of situations and mental states are composed of sums of representations of the actions they afford
Source: Nat Commun. 2024 Jan 19;15:620. doi: 10.1038/s41467-024-44870-7 (PMC10799018; doi:10.1038/s41467-024-44870-7)
Supplement: Supplementary file 3 — Reporting Summary [file 41467_2024_44870_MOESM3_ESM.pdf]

nature portfolio

Corresponding author(s): Mark A. Thornton

Last updated by author(s): Nov 30, 2023

Reporting Summary

Nature Portfolio wishes to improve the reproducibility of the work that we publish. This form provides structure for consistency and transparency in reporting. For further information on Nature Portfolio policies, see our Editorial Policies and the Editorial Policy Checklist.

Statistics

For all statistical analyses, confirm that the following items are present in the figure legend, table legend, main text, or Methods section.

n/a	Confirmed	
☐	☒	The exact sample size (*n*) for each experimental group/condition, given as a discrete number and unit of measurement
☐	☒	A statement on whether measurements were taken from distinct samples or whether the same sample was measured repeatedly
☐	☒	The statistical test(s) used AND whether they are one- or two-sided<br>*Only common tests should be described solely by name; describe more complex techniques in the Methods section.*
☐	☒	A description of all covariates tested
☐	☒	A description of any assumptions or corrections, such as tests of normality and adjustment for multiple comparisons
☐	☒	A full description of the statistical parameters including central tendency (e.g. means) or other basic estimates (e.g. regression coefficient) AND variation (e.g. standard deviation) or associated estimates of uncertainty (e.g. confidence intervals)
☐	☒	For null hypothesis testing, the test statistic (e.g. *F*, *t*, *r*) with confidence intervals, effect sizes, degrees of freedom and *P* value noted<br>*Give P values as exact values whenever suitable.*
☒	☐	For Bayesian analysis, information on the choice of priors and Markov chain Monte Carlo settings
☐	☒	For hierarchical and complex designs, identification of the appropriate level for tests and full reporting of outcomes
☐	☒	Estimates of effect sizes (e.g. Cohen's *d*, Pearson's *r*), indicating how they were calculated

Our web collection on statistics for biologists contains articles on many of the points above.

Software and code

Policy information about availability of computer code

Data collection	Data were collected using the PsychoPy package in Python 2.7.
Data analysis	Data were analyzed using Data Analysis Modules for Neuroimaging pipeline (https://github.com/PrincetonUniversity/prsonpipe). Using this pipeline, we applied SPM12 for slice time correction, DARTEL for head motion correction, unwarping, and normalization, and FSL for high pass filtering. The general linear model was fit to the fMRI data using SPM12 within the SPM12w wrapper (https://github.com/wagner-lab/spm12w). Inferential statistics were performed using analyzed using R 4.0.3, including the glmnet, pracma, and DescTools packages (code here: https://osf.io/qwd2k/).

For manuscripts utilizing custom algorithms or software that are central to the research but not yet described in published literature, software must be made available to editors and reviewers. We strongly encourage code deposition in a community repository (e.g. GitHub). See the Nature Portfolio guidelines for submitting code & software for further information.

Data

Policy information about availability of data

All manuscripts must include a data availability statement. This statement should provide the following information, where applicable:

- Accession codes, unique identifiers, or web links for publicly available datasets
- A description of any restrictions on data availability
- For clinical datasets or third party data, please ensure that the statement adheres to our policy

Raw MRI data from this investigation is available on the OpenNeuro (https://openneuro.org/datasets/ds004226/). Other data from this investigation has been

deposited on the Open Science Framework (https://osf.io/qwd2k/).

Research involving human participants, their data, or biological material

Policy information about studies with human participants or human data. See also policy information about sex, gender (identity/presentation), and sexual orientation and race, ethnicity and racism.

Reporting on sex and gender	The imaging sample consisted of 16 female and 12 male participants. The online DIAMONDS rating sample consisted of 177 female, 209 male, 4 other, 3 prefer not to state sex participants. The online affordance rating sample consisted of 290 female, 580 male, and 30 prefer not to state sex participants. No sex or gender based analyses were conducted because the sample sizes did not afford sufficient power, and because we did not preregister any related hypotheses.
Reporting on race, ethnicity, or other socially relevant groupings	The imaging sample consisted of 13 Asian, 9 White, 2 Black, 3 multiracial, 1 other participants (self-reported). We did not include race as a covariate in any analyses as it is not a potential confound in our study design.
Population characteristics	The mean age of the imaging sample was 20.61; age range = 18-36. The mean age of the DIAMONDS rating sample was = 37.01; age range = 19-81. The mean rating of the online affordance rating sample was 25.99; age range = 18-66
Recruitment	Participants in the imaging sample were recruited from the Princeton University area via SONA systems. Participants in the online samples were recruited from Amazon Mechanical Turk via Cloud Research. These samples were not representative of the US or global population, and thus likely reflect culturally-specific understandings of action affordances.
Ethics oversight	Princeton University Institutional Review Board

Note that full information on the approval of the study protocol must also be provided in the manuscript.

Field-specific reporting

Please select the one below that is the best fit for your research. If you are not sure, read the appropriate sections before making your selection.

☐ Life sciences ☒ Behavioural & social sciences ☐ Ecological, evolutionary & environmental sciences

For a reference copy of the document with all sections, see nature.com/documents/nr-reporting-summary-flat.pdf

Behavioural & social sciences study design

All studies must disclose on these points even when the disclosure is negative.

Study description	Quantitative, within-subject condition-rich neuroimaging design.
Research sample	Participants were recruited as convenience samples from the Princeton University area (imaging) and Amazon Mechanical Turk (online samples). The imaging sample consisted of 16 female and 12 male participants. The online DIAMONDS rating sample consisted of 177 female, 209 male, 4 other, 3 prefer not to state sex participants. The online affordance rating sample consisted of 290 female, 580 male, and 30 prefer not to state sex participants. The imaging sample consisted of 13 Asian, 9 White, 2 Black, 3 multiracial, 1 other participants (self-reported). The mean age of the imaging sample was 20.61; age range = 18-36. The mean age of the DIAMONDS rating sample was = 37.01; age range = 19-81. The mean rating of the online affordance rating sample was 25.99; age range = 18-66. These samples were selected due to their convenience (e.g., living close to the imaging site) are unlikely to be representative of the global population, and this places limits on the generalizability of the present findings.
Sampling strategy	Convenience sampling. The size of the imaging sample was determined using a parametric a priori power analysis based on the effect size of a prior study of similar design.
Data collection	The imaging sample completed the experiment in a 3T fMRI scanner. Participants were alone in the scanner room during the task, with only researchers in the control room outside. Neither the researchers nor the participants were blinded to the conditions, but all participants experienced all conditions intermixed, and did not communicate with the researchers while making their responses. The online samples completed their portions of the study on web surveys constructed for this purpose. All task administration was performed automatically.
Timing	The imaging sample was collected from December 13, 2019 to March 15, 2020. The DIAMONDS ratings were collected on May 29th, 2019. The online affordance ratings were collected on September 19, 2020.
Data exclusions	1 participant was excluded from the imaging study due to excessive head motion.
Non-participation	No participants withdrew from the imaging study, and withdraw from the online study could not be tracked.
Randomization	All condition randomization was conducted within-subject. The study designed was fully-crossed at the condition level, and partially crossed at the trial level.

Reporting for specific materials, systems and methods

We require information from authors about some types of materials, experimental systems and methods used in many studies. Here, indicate whether each material, system or method listed is relevant to your study. If you are not sure if a list item applies to your research, read the appropriate section before selecting a response.

Materials & experimental systems

n/a	Involved in the study	
☒	☐	Antibodies
☒	☐	Eukaryotic cell lines
☒	☐	Palaeontology and archaeology
☒	☐	Animals and other organisms
☒	☐	Clinical data
☒	☐	Dual use research of concern
☒	☐	Plants

Methods

n/a	Involved in the study	
☒	☐	ChIP-seq
☒	☐	Flow cytometry
☐	☒	MRI-based neuroimaging

Plants

Seed stocks	N/A
Novel plant genotypes	N/A
Authentication	N/A

Magnetic resonance imaging

Experimental design

Design type	Condition rich within subjects design
Design specifications	Participants completed 10 runs of this task over the course of the experiment. Each run consisted of 90 trials, including 30 situation-state pairs, 30 situation-action pairs, and 30 state-action pairs. Within each run, participants would see only 30 situations, 30 states, and 30 actions (twice each). This meant that the same situations, states, and actions were used evenly for each type of pairing (i.e., the 30 states paired with situations on a given run would also be the 30 states paired with actions on that run). This allowed us to strictly control how often each stimulus co-occurred with the two other classes (i.e., so that a certain action didn't occur more with situations than with mental states). Across pairs of runs (e.g., runs 1 and 2), the full set of 60 stimuli of each class would be presented. The sets of 60 were randomly divided in half independently for each pair of runs (e.g., the run 1-2 split would be different from the run 3-4 split) and across participants and stimulus classes. Thus, over the course of the 10 runs, each stimulus was guaranteed to be presented exactly 10 times. However, the particular pairings between stimuli of different classes were randomized within each run, subject to the constraint that no particular pairing was repeated within-subject. This produces a partially-crossed design at the level of pairings (i.e., there are 60 x 60 x 3 = 10,800 unique pairings between stimuli, but each imaging participant saw only 900 of them).
Behavioral performance measures	Participant responses and reaction times were recorded. Responses indicated how often participants thought different situations, mental states, and action co-occur.

Acquisition

Imaging type(s)	functional, structural
Field strength	3
Sequence & imaging parameters	Functional echo-planar BOLD images were collected with TR of 1500 ms; TE of 32 ms; flip angle of 70, and spatial resolution of 2.5 mm isotropic voxels. The preregistration called for 2 mm voxels, but after piloting, we decided that a larger voxel size produced better signal. Slices (52) were acquired in an interleaved, axial fashion with a simultaneously multislice acquisition factor of four. In addition to BOLD EPIs, we acquired a high-resolution anatomical image from each participant for the purposes of intersubject alignment. These images were generated by a T1-weighted scan with 1 mm isotropic voxels, a TR of 2300 ms, TE of 2.98 ms, flip angle of 9, and 176 slices. We also collected two spin echo field maps (phase encoding A >> P and P >> A) to correct for inhomogeneities in the magnetic field via unwarping. These

scans featured 2.5 mm isotropic voxels at a TR of 8000 ms and TE of 66 ms, with 52 transversal slices. A localizer and AA scout were used to determine the position of participants' brains and align scans accordingly.

Area of acquisition: Whole brain

Diffusion MRI: ☐ Used ☒ Not used

Preprocessing

Preprocessing software: Data Analysis Modules for Neuroimaging pipeline (https://github.com/PrincetonUniversity/prsonpipe). Using this pipeline, we applied SPM12 for slice time correction 60, DARTEL for head motion correction, unwarping, and normalization, and FSL for high pass filtering.

Normalization: DARTEL

Normalization template: MNI

Noise and artifact removal: Motion parameters

Volume censoring: None

Statistical modeling & inference

Model type and settings: First level GLMs, 2nd level pattern summation analyses and representational similarity analyses.

Effect(s) tested: No univariate statistical effects were tested.

Specify type of analysis: ☐ Whole brain ☒ ROI-based ☐ Both

Anatomical location(s): Reliability-based feature selection

Statistic type for inference: N/A (no wholebrain analyses)

(See Eklund et al. 2016)

Correction: Multiple comparisons were controlled via maximal statistic permutation testing.

Models & analysis

n/a	Involved in the study
☒	☐ Functional and/or effective connectivity
☒	☐ Graph analysis
☐	☐ Multivariate modeling or predictive analysis

Multivariate modeling and predictive analysis: Testing whether patterns associated with situations and mental states could be reconstructed from weighted sums of patterns associated with actions. Testing whether neural pattern similarity between situations, mental states, and actions could be predicted from action affordance ratings.
